# A *TTPA* deletion is associated with retinopathy with vitamin E deficiency in the English Cocker Spaniel dog

**DOI:** 10.1093/g3journal/jkaf016

**Published:** 2025-01-28

**Authors:** James A C Oliver, Katherine Stanbury, Ellen Schofield, Bryan McLaughlin, Cathryn S Mellersh

**Affiliations:** Ophthalmology Department, DWR Veterinary Specialists, Cambridgeshire CB8 0UH, UK; Canine Genetics Centre, Department of Veterinary Medicine, University of Cambridge, Cambridgeshire CB3 0ES, UK; Canine Genetics Centre, Department of Veterinary Medicine, University of Cambridge, Cambridgeshire CB3 0ES, UK; Canine Genetics Centre, Department of Veterinary Medicine, University of Cambridge, Cambridgeshire CB3 0ES, UK; Canine Genetics Centre, Department of Veterinary Medicine, University of Cambridge, Cambridgeshire CB3 0ES, UK

**Keywords:** inherited eye disease, genetics, ataxia with vitamin E deficiency, retinopathy, canine genetics, vitamin E, neurological abnormalities, genome-wide association study, whole genome sequencing

## Abstract

Retinopathy with vitamin E deficiency is a familial disease in the English Cocker Spaniel dog breed. Ophthalmic abnormalities observed in retinopathy with vitamin E deficiency-affected English Cocker Spaniel include lipofuscin granule deposition within the tapetal fundus and subsequent retinal degeneration resulting in visual deficits. Affected dogs may also exhibit neurological signs that include ataxia and hindlimb proprioceptive deficits. In all cases, circulating plasma concentrations of α-tocopherol are low. This study sought to investigate the genetic basis of retinopathy with vitamin E deficiency in the English Cocker Spaniel breed. We undertook a genome-wide association study comprising 30 English Cocker Spaniels with normal fundic examinations aged 6 years or older (controls) and 20 diagnosed with retinopathy with vitamin E deficiency (cases) and identified a statistically associated signal on chromosome 29 (*P*_raw_ = 1.909 × 10^−17^). Whole genome sequencing of 2 cases identified a 102 bp deletion in exon 1 of the alpha-tocopherol transfer protein gene (*TTPA*), truncating the protein by 34 amino acids. The c.23_124del variant segregated with retinopathy with vitamin E deficiency in a total of 30 cases and 43 controls. Variants in *TTPA* are causal for ataxia with vitamin E deficiency in humans which is a phenotypically similar disease to retinopathy with vitamin E deficiency. The identification of the canine variant is extremely significant as the availability of a DNA test will allow for identification of presymptomatic dogs and early therapeutic intervention which may prevent development of retinopathy and improve neurological signs. Breeders can also use the DNA test to efficiently eradicate the disease from this breed.

## Teaser

Retinopathy with vitamin E deficiency (RVED) is an inherited disease in the English Cocker Spaniel that causes blindness and ataxia. It has many similarities to ataxia with vitamin E deficiency (AVED) in humans. This study investigated the genetic basis of RVED and reveals it to be associated with a mutation in *TTPA* – the same gene that causes AVED. A DNA test has now been developed which will enable eradication of the disease from this dog breed and also allow for the identification of presymptomatic individuals. The latter would allow for early therapeutic intervention and prevention of retinal (and neurological) disease.

## Introduction

Vitamin E comprises 8 naturally occurring fat-soluble nutrients called tocopherols and tocotrienols. Vitamin E is an antioxidant that maintains cell membrane stability by prevention of lipid peroxidation (Drevon 1991; Herrera and Barbas 2001). Deficiency in vitamin E may result in pathologic changes in muscle, the reproductive tract, the central nervous system, and the retina (Hayes *et al*. 1970; McLellan *et al*. 2003; Stocker 2007; Traber and Head 2021). In dogs, dietary vitamin E deficiency leads to a multifocal pigmentary retinopathy (Riis *et al*. 1981; Davidson *et al*. 1998). Histologically, lipofuscin accumulation occurs within the retina and also within smooth muscle cells of the intestinal tract and within neurons of the CNS (Riis *et al*. 1981; Davidson *et al*. 1998).

A retinopathy with identical ophthalmoscopic signs to canine dietary vitamin E deficiency has been reported in several dog breeds including the Labrador Retriever, Golden Retriever, Briard, Border Collie, Polish Lowland Sheepdog, and English Cocker Spaniel (ECS) suggesting an inherited component in these breeds (Parry 1954; Barnett 1969; Aguirre and Laties 1976; McLellan *et al*. 2002; Bedford 2009). This retinopathy has been variably termed central progressive retinal atrophy, vitamin E deficiency retinopathy, and retinal pigment epithelial dystrophy (Parry 1954; Aguirre and Laties 1976; Riis *et al*. 1981; Lightfoot *et al*. 1996; Bedford 2009). Histologically, there is initial accumulation of lipofuscin within the retinal pigment epithelium followed by degeneration of the neurosensory retina characterized by a gradual loss of the outer nuclear layer and the subsequent atrophy and degeneration of the inner retina (Lightfoot *et al*. 1996; McLellan *et al*. 2003). Lipofuscin accumulation also occurs within smooth muscle cells throughout the body and also throughout the CNS (McLellan *et al*. 2003).

This retinopathy has been shown to be a familial disease in the ECS (McLellan *et al*. 2002). The age of onset is unknown as dogs tend to be presented for specialist examination quite late in the disease process, however, the mean (SD) age of affected ECS was 5.93 (2.19) years in 1 study (McLellan *et al*. 2002). Ophthalmoscopically, the disease is characterized by development of multifocal light brown pigment spots within the tapetal fundus (McLellan *et al*. 2002). Subsequently, these lesions coalesce to form patches and there is degeneration of the neurosensory retina manifested as retinal vascular attenuation and tapetal hyperreflectivity (McLellan *et al*. 2002). Affected dogs have low circulating plasma concentrations of α-tocopherol (α-Toc), the most abundant and biologically active form of vitamin E, in the absence of dietary deficiency or intestinal malabsorptive disease. In addition, a subsequent study of retinopathy with vitamin E deficiency reported that several affected ECS also had clinical signs of neurological dysfunction which included ataxia, proprioceptive deficits, abnormal spinal reflexes, and muscle weakness (McLellan *et al*. 2003).

In humans, inherited diseases resulting in vitamin E deficiency are most commonly associated with ataxia and occur as autosomal recessive disorders. To date, 2 forms of autosomal recessive ataxia due to vitamin E deficiency have been described. The first form to be described was abetalipoproteinemia in which there is a failure of chylomicron formation and absence of lipoproteins (Burnett *et al*. 1993). This leads to impaired gastrointestinal absorption and severely reduced plasma α-Toc concentrations. Abetalipoproteinemia is caused by mutations in *MTTP*—the gene encoding the microsomal triglyceride transfer protein (Sharp *et al*. 1993; Chardon *et al*. 2009). In the second form, ataxia with vitamin E deficiency (AVED), gastrointestinal absorption of lipids is normal but there is impaired incorporation of α-Toc into lipoproteins secreted by the liver (Traber *et al*. 1990). AVED is caused by mutations in *TTPA*—the gene encoding α-tocopherol transfer protein (Cavalier *et al*. 1998). Retinitis pigmentosa and pigmentary retinopathies have been associated with both forms making both *MTTP* and *TTPA* plausible candidate genes for retinopathy with vitamin E deficiency (RVED) (Matsuo *et al*. 1994; Yokota *et al*. 1996, 1997, 2000; Shimohata *et al*. 1998; Benomar *et al*. 2002; Mariotti *et al*. 2004; Ferreira *et al*. 2014; Iwasa *et al*. 2014; Nagappa *et al*. 2014; Abramowicz *et al*. 2024).

In this study, we investigated the molecular basis of RVED in the ECS using a combination of genome-wide association and whole genome sequencing strategies. Our ultimate aim was to develop a molecular test for breeders to use as a tool to eradicate the disease from the breed. A DNA test would also allow for the identification of young, presymptomatic individuals for early therapeutic intervention with potential avoidance of retinal and neurological disease.

## Materials and methods

### Sample collection

**Fig. 1.**
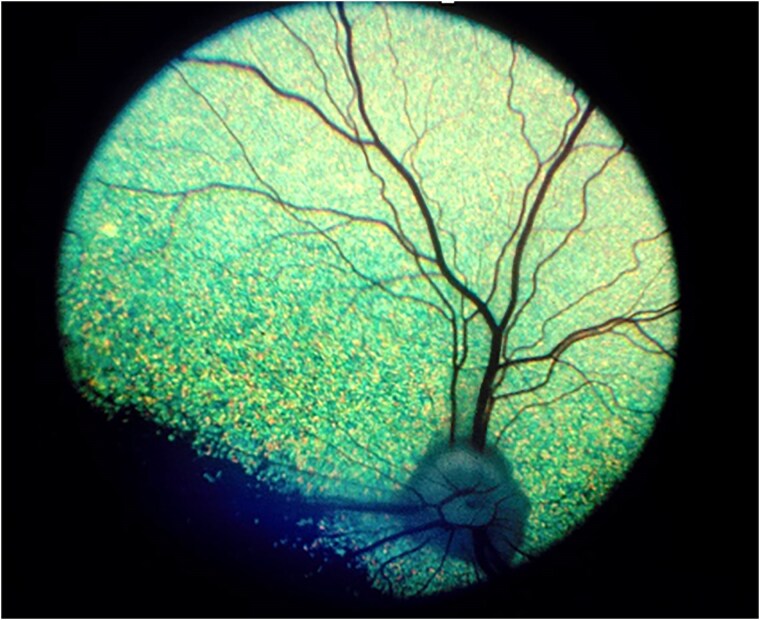
Fundus photograph of 1 RVED-unaffected English Cocker Spaniel included in this study (control).

**Fig. 2.**
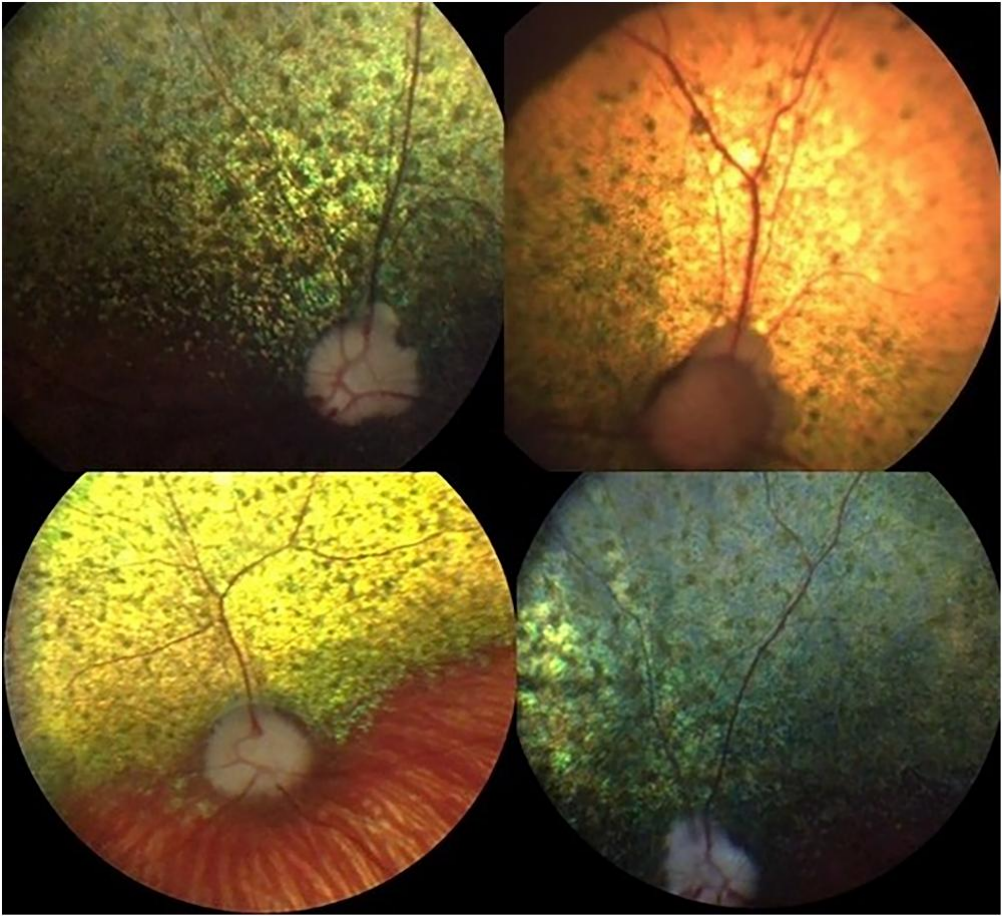
Fundus photographs of 4 RVED-affected English Cocker Spaniels with early retinopathy with vitamin E deficiency included in this study (cases). Multifocal gray-brown spots are present within the tapetal fundus of each dog representing lipofuscin granule deposition within the retinal pigment epithelium.

**Fig. 3.**
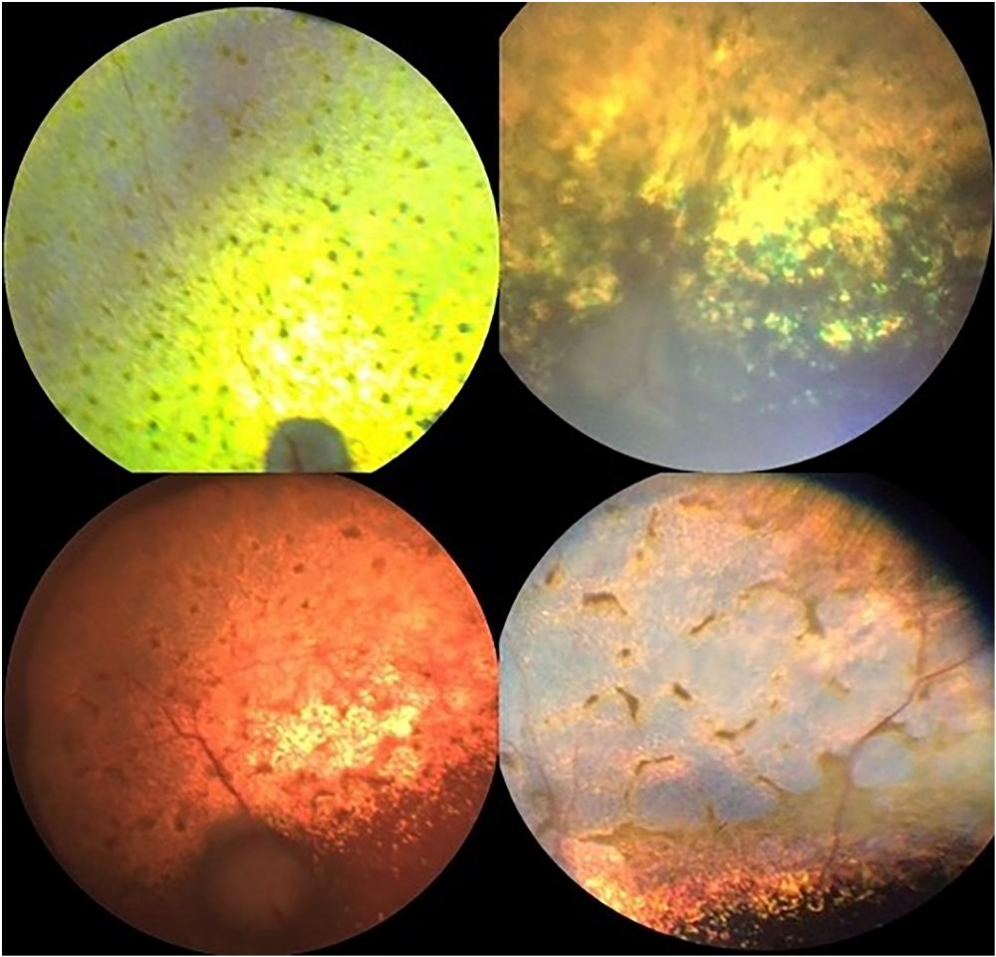
Fundus photographs of 4 RVED-affected English Cocker Spaniels with advanced retinopathy with vitamin E deficiency included in this study (cases). All cases show significant retinal degeneration characterized by retinal vascular attenuation and tapetal hyperreflectivity.

Dog owners and veterinary ophthalmologists submitted ECS DNA samples to the Canine Genetics Centre (previously based at the Animal Health Trust, Newmarket, UK) as buccal mucosal swabs or residual blood samples, with owner consent (ethical approval by Animal Health Trust Clinical Research Ethics Committee Project No. 24-2018E (2018) and University of Cambridge Department of Veterinary Medicine Ethics and Welfare Committee No. CR695 (2023) and CR496 (2021). DNA was extracted from both blood and buccal swabs using QIAamp DNA Blood Mini or Midi Kits (Qiagen, Manchester, UK). This study was performed in accordance with the ARVO Statement for Use of Animals in Research. Dogs were designated as RVED cases or controls following examination by board-certified veterinary ophthalmologists. The inclusion criteria for cases and controls were as follows:

1. Controls: ECS aged 6 years or older with no evidence of retinopathy on ophthalmoscopy (Fig. 1).
2. Cases:
  - RVED-affected cases. ECS presenting with owner-perceived visual deficits, ophthalmoscopic signs consistent with RVED, and plasma α-Toc concentrations < 20 μmol/l (Figs. 2 and 3). One case was reported to be an English Cocker Spaniel/Cavalier King Charles Spaniel (ECS/CKCS) cross.
  - RVED-suspected cases. ECS presenting with owner-perceived visual deficits, ophthalmoscopic signs consistent with RVED but for which plasma α-Toc concentrations were unavailable (Figs. 2 and 3).

### Ophthalmoscopy

Control and case status was established following ophthalmoscopy by a board-certified veterinary ophthalmologist. Following pharmacological mydriasis with 1% tropicamide, each eye of each dog was examined with both indirect and direct ophthalmoscopy (equipment varied between ophthalmologists). Control dogs had no evidence of retinopathy (Fig. 1) and cases had signs consistent with RVED based on previous published clinical descriptions of the disease (McLellan *et al*. 2003). Affected dogs are recognized clinically by the appearance of gray-brown pigment spots (Fig. 2), and subsequently patches, in the tapetal fundus. Degeneration of the neurosensory retina results in associated areas of increased tapetal reflectivity (Fig. 3).

### Genome-wide association study (GWAS)

Genotyping of 20 ECS RVED cases and 30 ECS controls was carried out using the Illumina CanineHD 230k array. The genome-wide association study (GWAS) data were analyzed for association using PLINK version 1.9 (Purcell *et al*. 2007). Quality control of data included the exclusion of SNPs with a minor allele frequency of <5% and missing genotype calls of >10% and the sample call rate for individuals was >99.7%. A multidimensional scaling plot was generated using PLINK to assess for the presence of population stratification. A plot of negative log (base 10) *P*-values was performed. A *P*-value of 0.05 after correction for multiple testing using the Bonferroni correction was the threshold for statistical significance. A test of SNPs in linkage disequilibrium (*r*^2^) with the top SNP from the GWAS was carried out in PLINK.

### Whole genome sequencing

Two ECS cases were selected for whole genome sequencing (WGS). Sequencing was outsourced to Edinburgh Genomics, United Kingdom where a TruSeq Nano 150 bp paired-end library was prepared and sequenced on the Illumina HiSeq X platform, generating approximately 30× genome coverage. Read data were aligned to the CanFam4 UU Cfam GSD 1.0 reference genome using BWA-MEM v0.7 (Li and Durbin 2009). Base quality score recalibration, indel realignment, and duplicate removal were performed using the Genome Analysis Toolkit (GATK) v4.2 according to GATK Best Practices recommendations (McKenna *et al*. 2010; Poplin *et al*. 2018). SNP/INDEL discovery was performed using GATK HaplotypeCaller (McKenna *et al*. 2010) and then loaded into a GenomicsDB. Joint variant calling was performed across 309 samples, including the 2 ECS cases, and the resulting variant calls filtered using standard hard-filtering parameters. The filtered variants were annotated and functional effects predicted using SnpEff v5.1 (Cingolani *et al*. 2012) and visualized in the Integrative Genomics Viewer (IGV) software (Robinson *et al*. 2011, 2022).

### WGS variant filtering

The first stage of variant filtering was performed using WGS of 307 dogs comprising 109 breeds and 2 cross breeds, via an in-house pipeline that scores variants based on the predicted effect on the protein. The 307 WGS consist of dogs with varying phenotypes excluding RVED, therefore, all 307 dogs acted as controls. Pedigree analysis of RVED cases indicated a recessive mode of inheritance for the disease and, therefore, a criterion of the variant filtering was that both cases had to be homozygous for an alternate allele and controls either heterozygous or homozygous for the reference allele. Variants retained after the first stage of filtering, with the highest effect score and homozygous in both cases, were then further filtered against a Variant Call Format (VCF) file containing 1987 WGS consisting of 1,611 dogs (321 breeds), 309 village dogs, 63 wolves, and 4 coyotes curated by the Dog10K Consortium (Meadows *et al*. 2023).

### Genotyping of WGS filtered variant by Sanger sequencing and amplified fragment length polymorphism (AFLP)

After the exclusion of common (present in multiple canine breeds) variants, a single variant remained after filtering which was homozygous in both RVED-affected ECS. The variant was a deletion located in exon 1 of *TTPA*. The variant was verified initially by Sanger sequencing of 2 cases and 2 controls. PCR products were amplified using HotstarTaq DNA Polymerase (Qiagen), 1.5 mM 60:40 d7GTP:GTP dNTP mix and Q Solution (Qiagen). Cycling conditions were: 98°C for 15 min; 35 cycles at 98°C for 30 s; 59°C for 30 s; 72°C for 30 s; and 72°C for 5 min. Amplified products were sequenced in both directions at Source Bioscience, Cambridge, United Kingdom. Sequence traces were analyzed using the Staden software package (Staden *et al*. 2000). Further variant validation was carried out by amplified fragment length polymorphism (AFLP) in 30 cases and 43 controls. PCR products were amplified using HotstarTaq DNA Polymerase (Qiagen), 1.5 mM 60:40 d7GTP:GTP dNTP mix, Q Solution (Qiagen), a FAM fluoresced tailed forward primer (Supplementary Table 2). Cycling conditions were: 98°C for 15 min; 35 cycles at 95°C for 30 s; 59°C for 30 s; 72°C for 1 min and then 8 cycles at 94°C for 30 s; 50°C for 30 s, 72°C for 1 min and then 72°C for 30 min. Primers were designed using Primer3 (Untergasser *et al*. 2012) (Supplementary Table 2) to flank the deletion. Amplified products were outsourced to the Department of Biochemistry, University of Cambridge, United Kingdom, for AFLP using an ABI 3130xl DNA Analyzer (Applied Biosystems). Fragment length analysis was then carried out using Genemarker v.3.0.1 (Softgenetics LLC, USA).

A further 186 ECS were genotyped by AFLP as above to ascertain the variant frequency.

## Results

### RVED cases

A total of 21 RVED-affected and 9 RVED-suspected ECS samples were analyzed for this study (Supplementary Table 1). Five-suspected RVED cases reportedly also had low circulating plasma α-Toc concentrations but laboratory results were unavailable to confirm this. The mean age at diagnosis of the cases was 5.28 years and the median age, 5 years. The mean α-Toc plasma concentration where levels were provided (*n* = 21) was 5.65 μmol/l and the median 3.3 μmol/l. Two cases aged 3.17 and 6.67 years at diagnosis showed neurological clinical signs of hindlimb proprioceptive deficits with the latter also observed to have hindlimb ataxia. One case aged 3 years suffered from seizures. Facial nerve paresis was reported in a 7-year-old ECS case. Pedigree analysis indicated that the disease segregates with a recessive mode of inheritance. Figure 1 shows a fundus photograph of one of the control dogs. Figures 2 and 3 illustrate fundic disease presentation of RVED at early and late stages of the disease, respectively.

### GWAS

**Fig. 4.**
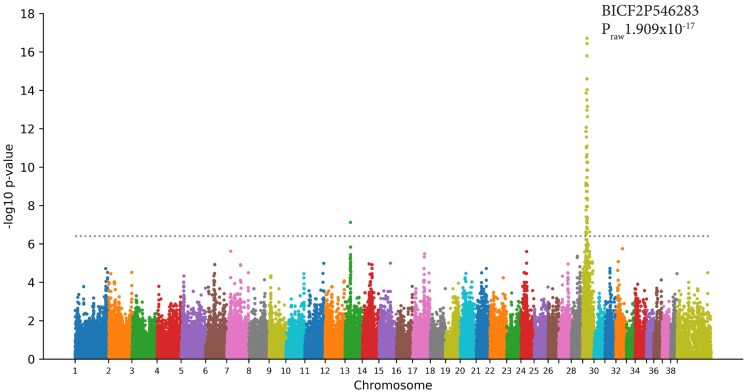
Genome-wide association analysis of RVED in English Cocker Spaniels. Manhattan plot of association of RVED in ECS is shown. Genome-wide association significance is determined by Bonferroni correction shown by the dotted line. The top SNP is shown with the log_10_*P*_raw_ value. All GWAS SNPs are based on the CanFam3.1 reference genome.

After quality control, the genome-wide association analysis was carried out using genotyping data containing 127,557 SNPs, 18 cases, and 30 controls. A strong statistical signal was observed on chromosome 29 (*P*_raw_ 1.909 × 10^−17^) exceeding the Bonferroni threshold −log10 *P*-value of 6.41 (Fig. 4). A second signal exceeding the Bonferroni threshold occurs on chromosome 13 (*P*_raw_ 7.42 × 10^−8^). A region containing SNPs that were in linkage disequilibrium with the top SNP (BICF2P546283) (i.e. with an *r*^2^ value of >0.80) spanned a region of approximately 4.7 Mb from SNP chr29_13257379 to BICF2S23527829 (chr29:13257379–16318933 based on CanFam3.1).

**Fig. 5.**
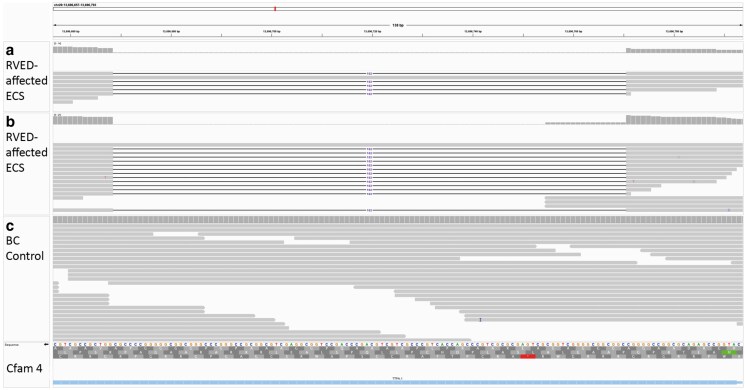
WGS reads in IGV of RVED-affected ECS and a control RVED-unaffected Border Collie dog. WGS reads are based on the canine CanFam4 UU Cfam GSD 1.0 reference genome. a) and b) illustrate local alignments generated using GATK HaplotypeCaller using the genomic co-ordinates produced by the VCF of the 2 ECS RVED cases (Poplin *et al*. 2018). The 102 bp deletion is illustrated in a) and b) with black lines joining alignments annotated with deletion size. Reads in a) and b) without the 102 bp annotation are representative of artificial haplotypes created by HaplotypeCaller and based on the reference sequence. Reads shown in c) are from an RVED-unaffected Border Collie control WGS. The 102 bp deletion is located in exon 1 of *TTPA* (chr29:13,696,668–13,696,771).

**Fig. 6.**
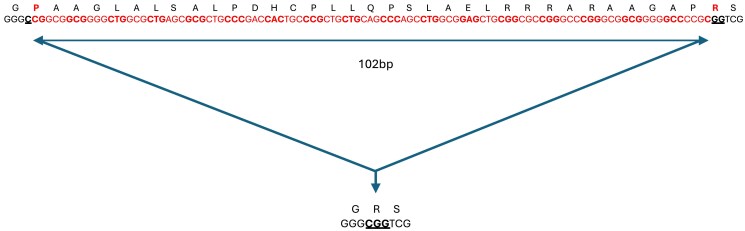
Sequence of 102 bp deletion identified in exon 1 of *TTPA*. The 102 bp nucleotide deletion is shown in red with the amino acid translation above. The deletion remains in frame. It commences at p.P8 in the last 2 nucleotides of the codon and ends with the first nucleotide of p.R42. The nucleotides flanking the deletion (underlined) result in a missense amino acid change from P > R at p.8 and the sequence then continues as per the wild-type sequence from position p.43.

Twenty-two variants with the highest effect on the protein were homozygous in the 2 ECS RVED-affected WGS. Only 1 variant was private to the cases after filtering against our in-house 307 WGS and the Dog10K VCF (Meadows *et al*. 2023). The variant was a 102 bp deletion located in exon 1 of *TTPA*, the gene that encodes the alpha-tocopherol transfer protein (Fig. 5). The deletion truncates the protein by 34 amino acids but remains in frame (Fig. 6).

### Potential pathogenicity of c.23_124del *TTPA* variant

**Fig. 7.**
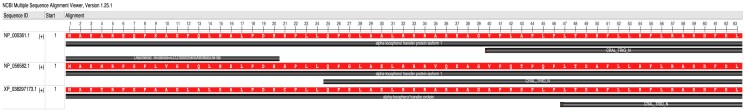
Human, canine, and mouse α-TTP protein alignment. The α-TTP amino acid alignment between human (NP_000361.1), mouse (NP_056582.1), and dog (XP_038297173.1) shows predicted functional domains of the protein. Amino acids are colored in red indicating that this is a highly conserved region (based on the relative entropy threshold of the residue). The deletion removes amino acids from position p.8 to p.42. The CRAL-TRIO-N lipid-binding domain in the mouse and human would be affected in addition to the Disordered domain in the human. The CRAL-TRIO domain commences at p.47 in the dog and is, therefore, not predicted to be affected. The alignment was carried out using the National Center for Biotechnology Information Constraint-based Multiple Alignment Tool version 1.25.1 and colored using the “Conservation” method (Papadopoulos and Agarwala 2007).

**Fig. 8.**
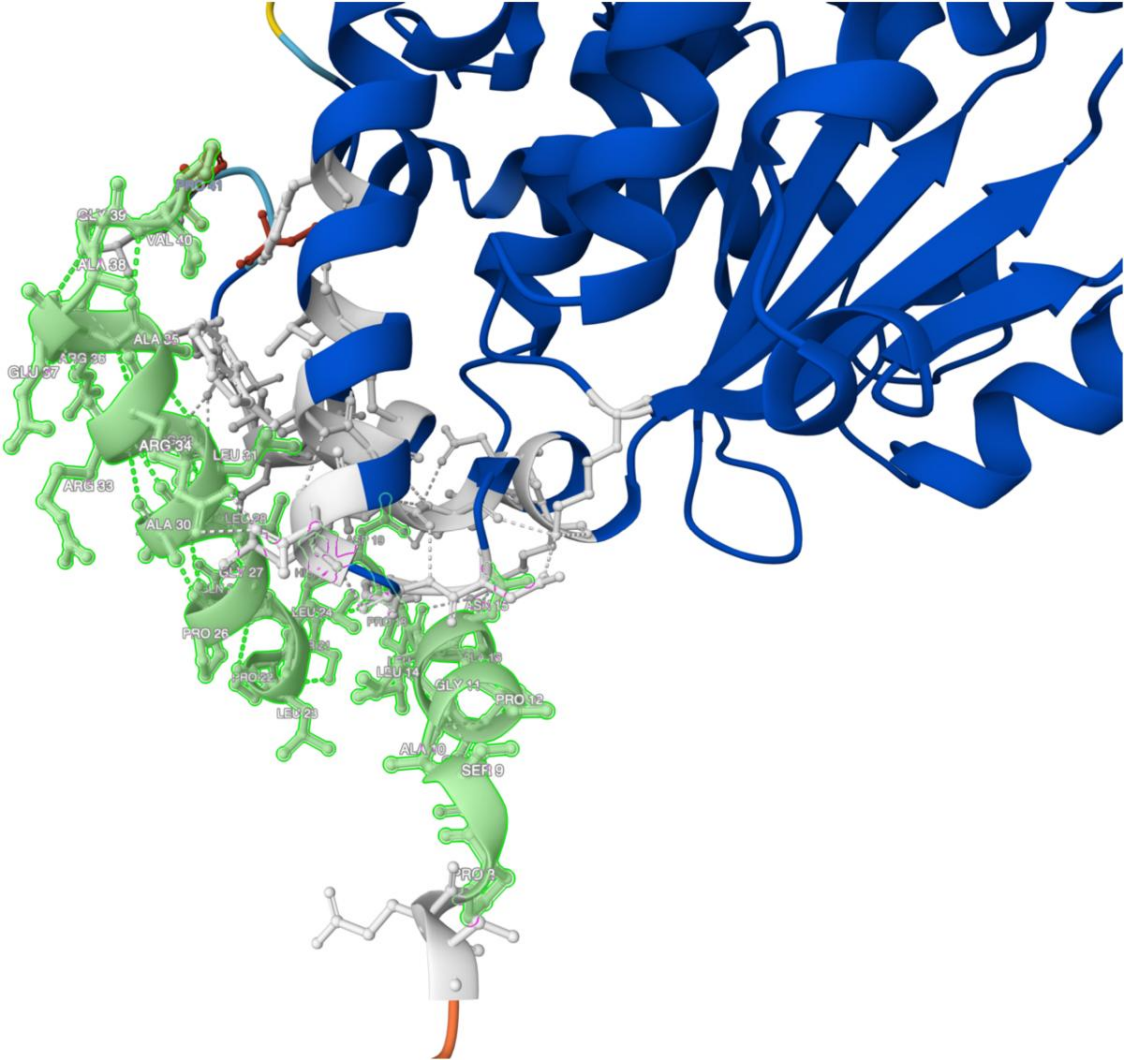
AlphaFold prediction of the 3D α-TTP protein structure in the human. The graphic shown in this figure is the predicted α-TTP protein configuration in the human generated by AlphaFold (Jumper *et al*. 2021; Varadi *et al*. 2021; Varadi *et al*. 2023). The per-residue model confidence score is “low” (pLDDT 0–70) from p.1 to p.10, “high” from p.11 to p.13 (pLDDT 70–90), and “very-high” (pLDDT > 90) from position p.14. The region annotated by amino acid position and shown in green is the 34 amino acids deleted from the ECS RVED cases. The interactions of deleted amino acids with surrounding amino acids are shown in gray and illustrate their relationship with the adjacent α-helix. Dotted lines represent hydrogen bonds between amino acids.

**Fig. 9.**
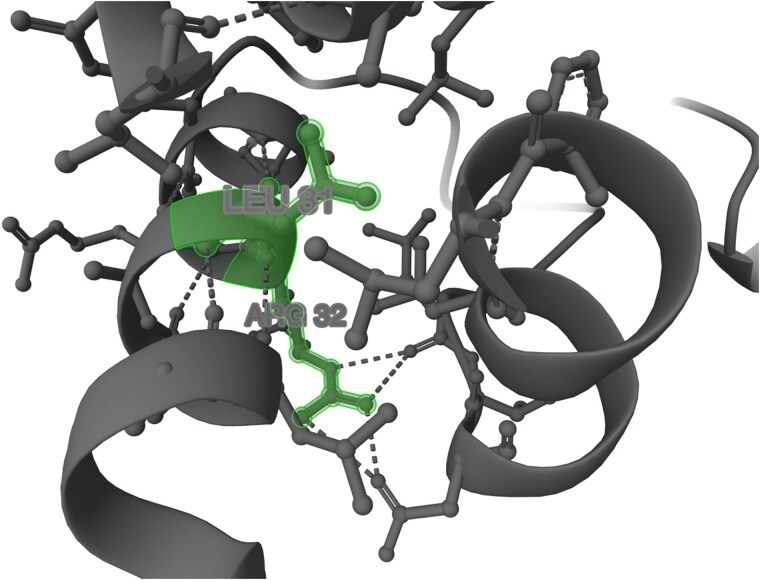
AlphaFold 3D human α-TTP protein centered on p.L31 and p.R32. Protein positions p.L31 and p.R32 are shown highlighted in green. AlphaFold predicts that in the human, any amino acid alteration at these locations would be pathogenic (Minton 2023).

To ascertain the potential pathogenicity of the c.23_124del *TTPA* variant, we analyzed the level of conservation of deleted amino acids and searched for annotated functional domains within the protein in the human, mouse, and dog (Fig. 7). We also evaluated the effects the variant may have on the protein structure using AlphaFold (Figs. 8 and 9) (Jumper *et al*. 2021).

### *TTPA* variant validation

The c.23_124del variant in *TTPA* was validated in a cohort of 30 ECS RVED cases and 43 ECS controls using AFLP. The variant segregated correctly with the disease with all cases homozygous for the 102 bp deletion. The results are shown in Table 1. The variant frequency among the clinically unaffected controls (all heterozygous for the deletion) was 0.09. Further genotyping by AFLP was carried out in a cohort of 186 ECS of unknown health status to ascertain the frequency of the variant (Table 2). The variant frequency in this cohort was 0.11 which is comparable to the clinically unaffected control cohort of ECS (Table 1).

**Table 1.** Results of amplified fragment length polymorphism analysis of cases and controls for the *TTPA* deletion.

Disease status	Homozygous deletion, del/del	Heterozygous deletion, WT/del	Homozygous wild-type, WT/WT	Total
RVED cases	30	0	0	30
Controls	0	8	35	43

**Table 2.** Results of amplified fragment length polymorphism for the *TTPA* deletion in 186 ECS randomly selected from an archived collection.

	Homozygous deletion, del/del	Heterozygous deletion, WT/del	Homozygous wild-type, WT/WT	Total number of ECS
Number of ECS	7	26	153	186

Seven ECS were homozygous for the variant and were subsequently followed up to ascertain the health status of each dog. The results of the follow-up are shown in Table 3.

**Table 3.** Health status of dogs homozygous for the 102 bp *TTPA* deletion after AFLP analysis.

Dog number	Health status	Age submitted
1	Suspected neuronal ceroid-lipofuscinoses (NCL), blind	∼3 years
2	Anterior subcapsular cataract (left eye)	0.75 years
3	Suspected progressive retinal atrophy (veterinarian submission)	6.5 years
4	Early signs of retinal degeneration	3 years
5	Unknown status	N/A
6	Owner submitted as “clear” of disease	0.2 years
7	Owner submitted as “clear” of disease	3.5 years

## Discussion

In this study, we employed a combined approach of GWAS and WGS to identify a 102 bp deletion in *TTPA* that is associated with RVED in the ECS. The variant was identified by WGS pipeline analysis of 2 cases aligned to the canine reference genome, CanFam4. The region identified in the GWAS (Fig. 4) strongly suggested an association with RVED on chromosome 29. The SNPs in linkage disequilibrium with the top SNP spanned a region that commenced at chr29: 13257379 which is within *YTHDF3*, upstream of *TTPA*. It was interesting to note that the top SNP was not located within *TTPA* itself. However, the SNPs on the Illumina CanineHD 230k array are all based on CanFam3.1 co-ordinates, a version of the reference genome in which *TTPA* is incorrectly annotated. There was an additional SNP that exceeded the Bonferroni threshold located on chromosome 13. Homozygosity mapping of RVED cases and controls did not reveal potential blocks of homozygosity present in all cases. Two RVED-affected ECS, however, did have a homozygous region on chromosome 13 surrounding the most associated SNP (data not shown). Visual interrogation of WGS reads of the 2 RVED-affected ECS in IGV in conjunction with WGS pipeline analysis did not reveal anything of significance in this region. We postulate, therefore, that the SNP on location 13 is specific to 2 individual cases and not related to RVED.

*TTPA* is an excellent candidate gene for RVED because mutations in this gene have been reported to cause a similar phenotype in humans. In humans, *TTPA* mutations are responsible for AVED, a disorder previously known as familial isolated vitamin E deficiency (FIVE) (Schuelke 1993; Hoshino *et al*. 1999; Alex *et al*. 2000; Cellini *et al*. 2002; Mariotti *et al*. 2004; Bouhlal *et al*. 2008; Di Donato *et al*. 2010; Euch-Fayache *et al*. 2014; Elkamil *et al*. 2015; Zea Vera *et al*. 2021; Zhang *et al*. 2022). Initial signs of AVED include progressive ataxia, clumsiness of the hands, loss of proprioception, and areflexia (Schuelke 1993). Retinitis pigmentosa also appears fairly common in AVED patients (Yokota *et al*. 1996, 1997, 2000; Shimohata *et al*. 1998; Pang *et al*. 2001; Iwasa *et al*. 2014; Abramowicz *et al*. 2024). In our study, all affected ECS had pigmentary retinopathy, although neurological disturbance was only reported in 3 dogs. A fourth dog was reported to have facial nerve paresis, however, it is uncertain as to whether this is associated with low plasma vitamin E (Cameron *et al*. 2007). It is likely that ataxia is more common in dogs with RVED than our study may suggest. A previous study of RVED in the ECS in which all dogs were examined physically, ophthalmologically, and neurologically reported neurological dysfunction to be common (McLellan *et al*. 2003). Eleven of 15 dogs (73%) showed signs including ataxia, proprioceptive deficits, abnormal spinal reflexes, and muscle weakness. Sample collection in our study was biased. All samples were recruited from veterinary ophthalmologists who were presented cases, following owner perception of visual deficits in their dogs. Had samples also been recruited from veterinary neurologists, more cases of RVED with ataxia may have been identified. It is possible that dog 1 (Table 3) that was suspected to be a neuronal ceroid-lipofuscinoses case but that was in fact homozygous for the *TTPA* c.23_124del variant is an example of such a case. Furthermore, although retinopathy was the most obvious presenting clinical sign in the affected dogs, no dogs underwent thorough neurological examination and so signs of subtle neurological dysfunction may have been missed.

*TTPA* encodes the α-tocopherol transfer protein (α-TTP) which is the only known protein to specifically bind α-Toc—the most abundant and biologically active form of vitamin E in higher animals (Arai and Kono 2021). α-TTP is highly expressed in the liver where α-TTP selects α-Toc taken up via plasma lipoproteins and promotes its secretion to circulating lipoproteins (Traber and Kayden 1989; Traber *et al*. 1990, 1992). Thus, α-TTP is a major determinant of plasma α-Toc concentrations. Although, α-TTP is highly expressed and has an important function in the liver, it is also expressed in the lung, spleen, uterus, brain, and retina (Hosomi *et al*. 1998; Kaempf-Rotzoll *et al*. 2002; Shichiri *et al*. 2012). α-TTP mRNA is detected predominantly in the Purkinje layer of the cerebellar cortex and, in AVED patients, loss of α-TTP causes severe damage to Purkinje cells in the brain (Larnaout *et al*. 1997; Hosomi *et al*. 1998; Ulatowski *et al*. 2014). α-TTP is expressed in Müller cells of the retina which may facilitate the transport of α-Toc from the blood capillaries to photoreceptor neurons (Shichiri *et al*. 2012). The photoreceptor outer segment membrane contains unusually high amounts of polyunsaturated fatty acids, which makes the membrane more susceptible to oxidation (Neuringer *et al*. 1988). In addition to its role in preventing lipid peroxidation, α-Toc may also protect oxidation of vitamin A which is essential to the visual process (Robison *et al*. 1979, 1980). α-Toc may also have a role in maintaining photoreceptor membrane fluidity which is necessary for the normal movement of rhodopsin molecules during phototransduction (Moran *et al*. 1987; Goss-Sampson *et al*. 1991).

In both dogs and humans, *TTPA* comprises 5 exons which encode a 278 amino acid translated product. To date, over 20 deleterious variants in *TTPA* have been reported to be associated with AVED in humans. Variants have been found in each of the 5 exons (Amiel *et al*. 1995; Gotoda *et al*. 1995; Ouahchi *et al*. 1995; Yokota *et al*. 1996, 1997, 2000; Cavalier *et al*. 1998; Shimohata *et al*. 1998; Hoshino *et al*. 1999; Usuki and Maruyama 2000; Di Donato *et al*. 2010). Variant type broadly correlates with both the age of onset and severity of clinical signs with truncations, frame-shift variants, and non-conserved substitutions resulting in more severe and early onset forms of AVED, in association with dramatic reductions in plasma α-Toc concentrations (Amiel *et al*. 1995; Hentati *et al*. 1996; Krendel *et al*. 1987; Roubertie *et al*. 2003; Mariotti *et al*. 2004). To the authors’ knowledge, the variant we report is the only naturally occurring mutation in *TTPA* in a nonhuman species. The mutation is a 102 bp deletion in exon 1 of *TTPA* which is predicted to result in a protein that is truncated by 34 amino acids and which presumably leads to loss of function, accounting for the severe reduction in plasma α-Toc concentrations and retinopathy, reported to occur in ECS with RVED. The deletion was private to the RVED cases after WGS filtering, however, it was subsequently identified in the UCSC Genome Browser (CanFam4) (Nassar *et al*. 2023). It was annotated whilst creating the structural variation track as part of the GSD_1.0/CanFam 4 reference assembly (Wang *et al*. 2021). The track was created from 10× sequencing data of 27 dogs of 19 breeds. Further investigation found that of the 27 dogs, 1 ECS was homozygous for the variant. Follow-up of the dog revealed that it had unilateral glaucoma but funduscopy was not performed and so RVED status is unknown. One RVED case was reported by the owner to be an ECS/Cavalier King Charles Spaniel cross. The dog was homozygous for the *TTPA* deletion variant. Genetic verification of the cross was not provided and, therefore, we postulate that as the variant was not detected in other dog breeds that this RVED case was not an F1 cross (ECS × Cavalier King Charles Spaniel) but rather an F2 cross with ECS on both the sire and dam's sides of its pedigree.

The potential effects that the c.23_124del variant may exert on the structure of the α-TTP protein in the human are shown in Figure 8. The first 3 α-helices would be deleted and thus would not provide structural support to surrounding structures. We can infer that this occurrence is analogous in the canine TTPA protein as amino acids in this region are highly conserved between the 2 species (Fig. 7). Two amino acids located at p.L31 and p.R32 are highlighted in Figure 9, both of which are highly conserved in mammals (Supplementary Fig. 1). AlphaFold predicts a change of amino acid (any amino acid) at these locations would be deleterious (Fig. 9). Amino acid p.L31 is maintaining the first α-helix structure, and amino acid p.R32 forms hydrogen bonds with p.L47 and p.D49. AlphaFold computes an average missense pathogenicity of any amino acid alteration at these locations to be 0.797 and 0.710, respectively, and are, therefore, predicted to be pathogenic (Minton 2023). We postulate, therefore, that deletion of these amino acids would also be pathogenic in the ECS and disrupt the protein structure. In the canine protein (Fig. 7), conserved domains are not predicted to be affected by the c.23_124del variant. However, in both the human and mouse, the CRAL-TRIO-N domain would be impacted. This is an important domain that facilitates interactions with bound lipid head groups (Li *et al*. 2023). Furthermore, in the human protein, the intrinsically disordered region would be removed as a result of the c.23_124del variant. Disordered regions are malleable and thus do not conform to a rigid 3D structure, they are important for biological processes such as cell signaling and subcellular organization (Holehouse and Kragelund 2024). This domain is not annotated in the dog but it is unknown as to whether this is a consequence of a lack of functional and computation analysis of the canine protein or whether it is truly absent.

RVED-affected dogs have been reported to have mean plasma α-Toc concentration of 3.78 μmol/l compared to 67.11 μmol/l in unaffected dogs (McLellan *et al*. 2002). This is consistent with the median concentration of 3.3 μmol/l in the cases in our study, and in which the mean α-Toc was 5.65 μmol/l. AVED is treated with vitamin E supplementation (Meydani *et al*. 1998). Treatment results in cessation of progression of signs of neurological dysfunction in most patients and improvement of signs in some (Yokota *et al*. 1996; Gohil and Azzi 2008; Kohlschütter *et al*. 2020). Favorable response to treatment relates to severity and duration of clinical signs before treatment is implemented. Treatment of presymptomatic individuals has also prevented development of AVED further underlying the importance of prompt treatment and also early identification of those at risk patients through molecular genetic testing (Schuelke 1993). In dogs with RVED, oral supplementation with vitamin E restored plasma α-Toc concentrations to within the normal reported range and appeared to halt progression of neurological disease, improve neurological signs, and exercise tolerance but not lead to resolution of pre-existing ocular signs (McLellan *et al*. 2002, 2003; McLellan and Bedford 2012). To date, it has not been possible to identify affected dogs until the onset of consistent clinical signs in association with severe reduction in plasma α-Toc concentrations. Now that a variant associated with RVED has been identified, a molecular DNA test can be developed which will both allow identification of presymptomatic individuals for therapeutic intervention along with those that carry the mutation. With appropriate use of DNA testing, breeders will be able to eradicate RVED from the breed efficiently. The ECS is a popular breed of dog in the United Kingdom with approximately 26,000 dogs being registered with the Kennel Club each year (Kennel Club data). Considering an estimated variant frequency of 0.09 and assuming random segregation of the variant within the population, this would equate to approximately 210 Kennel Club-registered ECS being homozygous for the mutation and thus affected with RVED each year. This itself is likely to be a gross underestimate of the total number of ECS affected by RVED in the United Kingdom, as only a minority of ECS are registered with the Kennel Club.

In conclusion, we have identified a deletion in *TTPA* that is associated with RVED in ECS. This appears to be the only spontaneously occurring mutation in *TTPA* in a nonhuman species and, as in humans with AVED, occurs as an autosomal recessive trait.

## Supplementary Material

jkaf016_Supplementary_Data

## Data Availability

Whole genome sequencing data for this study have been deposited in the European Nucleotide Archive (ENA) at EMBL-EBI under accession numbers: PRJEB79956 and PRJEB36029. BioSample accessions for the 2 RVED cases are SAMEA7189992 and SAMEA7190009. Supplemental material available at G3 online.
