# Supplementary material for: A TTPA deletion is associated with retinopathy with vitamin E deficiency in the English Cocker Spaniel dog
Source: G3 (Bethesda). 2025 Jan 28;15(4):jkaf016. doi: 10.1093/g3journal/jkaf016 (PMC12005162; doi:10.1093/g3journal/jkaf016)
Supplement: jkaf016_Supplementary_Data [file jkaf016_supplementary_data.zip › S1_Figure_G3-2024-405446.pdf]

NCBI Multiple Sequence Alignment Viewer, Version 1.25.2

| Sequence ID | Start | 148 | 149 | 150 | 151 | 152 | 153 | 154 | 155 | 156 | 157 | 158 | End | Organism |
|---|---|---|---|---|---|---|---|---|---|---|---|---|---|---|
| NP_000361.1 (+) | 1 | P | G | L | A | A | L | R | R | R | A | R | 278 | Homo sapiens |
| NP_056582.1 (+) | 1 | P | G | L | A | E | L | R | R | R | V | Q | 278 | Mus musculus |
| NP_037180.1 (+) | 1 | P | G | L | A | E | L | R | R | R | A | Q | 278 | Rattus norvegicus |
| NP_001185811.1 (+) | 1 | P | S | L | A | E | L | R | R | R | T | G | 282 | Ovis aries |
| NP_001193605.1 (+) | 1 | P | S | L | A | E | L | R | R | R | A | G | 282 | Bos taurus |
| XP_023504306.1 (+) | 1 | P | S | L | A | E | L | R | R | R | A | R | 278 | Equus caballus |
| XP_038297173.1 (+) | 1 | P | S | L | A | E | L | R | R | R | A | R | 278 | Canis lupus familiaris |
| XP_001092014.1 (+) | 1 | P | G | L | A | E | L | R | R | R | A | R | 278 | Macaca mulatta |
| XP_054512761.1 (+) | 1 | P | G | L | A | A | L | R | R | R | A | R | 317 | Pan troglodytes |
| XP_004773001.1 (+) | 1 | P | S | L | A | E | L | R | R | R | A | R | 278 | Mustela putorius furo |
| XP_034524010.1 (+) | 1 | P | S | L | A | E | L | R | R | R | A | R | 278 | Ailuropoda melanoleuca |
| XP_014440533.1 (+) | 1 | P | G | L | A | E | L | R | R | R | A | Q | 278 | Tupaia chinensis |
| XP_005066277.1 (+) | 1 | P | G | L | R | E | L | R | L | R | A | E | 278 | Mesocricetus auratus |
| XP_004842188.1 (+) | 1 | P | G | L | A | E | L | R | R | R | A | R | 278 | Heterocephalus glaber |
| XP_002758987.1 (+) | 1 | P | G | L | A | E | L | R | R | R | A | R | 278 | Callithrix jacchus |
| XP_044513926.1 (+) | 1 | P | G | I | R | E | L | R | K | R | T | E | 279 | Gracilinanus agilis |
| XP_001379317.1 (+) | 1 | P | G | I | R | E | L | R | K | R | A | E | 279 | Monodelphis domestica |
| XP_043832433.1 (+) | 1 | P | G | I | S | E | L | R | R | Q | A | E | 279 | Dromiciops gliroides |
| XP_051826091.1 (+) | 1 | S | G | I | S | E | L | R | Q | R | A | E | 279 | Antechinus flavipes |
| XP_003759792.1 (+) | 1 | S | G | I | S | E | L | R | Q | R | A | E | 279 | Sarcophilus harrisii |
| XP_020857847.1 (+) | 1 | P | G | I | S | E | L | R | R | R | A | E | 279 | Phascolarctos cinereus |
| XP_036608344.1 (+) | 1 | P | G | I | S | E | L | R | R | R | A | E | 279 | Trichosurus vulpecula |
| XP_040851884.1 (+) | 1 | P | G | L | T | E | L | R | R | R | A | L | 277 | Ochotona curzoniae |
| XP_058524651.1 (+) | 1 | P | G | L | A | E | L | R | R | R | A | L | 277 | Ochotona princeps |
| XP_062044424.1 (+) | 1 | P | G | L | A | E | L | R | R | R | A | Q | 278 | Lepus europaeus |
| XP_002710548.1 (+) | 1 | P | G | L | A | E | L | R | R | R | A | Q | 278 | Oryctolagus cuniculus |
| XP_048214630.1 (+) | 1 | P | G | V | Q | E | L | R | R | R | A | R | 301 | Perognathus longimembr... |
| XP_042522409.1 (+) | 1 | P | G | L | Q | E | L | R | R | R | A | R | 277 | Dipodomys spectabilis |
| XP_020037880.1 (+) | 1 | P | G | L | A | E | L | R | R | R | A | R | 278 | Castor canadensis |
| XP_021015863.1 (+) | 1 | P | G | L | A | E | L | R | R | R | V | Q | 278 | Mus caroli |
| XP_021078015.1 (+) | 1 | P | G | L | A | E | L | R | R | R | A | Q | 278 | Mus pahari |
| XP_052032096.1 (+) | 1 | P | G | L | A | E | L | R | R | R | A | Q | 278 | Apodemus sylvaticus |
| XP_042126608.1 (+) | 1 | | | | D | V | I | S | T | R | G | H | 283 | Peromyscus maniculat... |
| XP_052573875.1 (+) | 1 | P | G | L | A | E | L | R | R | R | V | E | 278 | Peromyscus californicus ... |
| XP_045000330.1 (+) | 1 | P | S | L | A | E | L | R | R | R | A | L | 278 | Jaculus jaculus |
| XP_060242024.1 (+) | 1 | P | G | L | A | E | L | R | R | R | A | Q | 278 | Meriones unguiculatus |
| XP_055459237.1 (+) | 1 | P | G | L | A | E | L | R | R | R | A | Q | 278 | Psammomys obesus |
| XP_051016757.1 (+) | 1 | P | G | L | A | E | L | R | R | R | A | L | 278 | Acomys russatus |
| XP_032761215.1 (+) | 1 | P | G | L | A | E | L | R | R | R | A | Q | 332 | Rattus rattus |
| XP_028636123.1 (+) | 1 | P | G | L | A | E | L | R | R | R | A | L | 278 | Grammomys surdaster |
| XP_031222381.1 (+) | 1 | P | G | L | V | E | L | R | R | R | A | Q | 296 | Mastomys coucha |
| XP_034346116.1 (+) | 1 | P | G | L | A | E | L | R | R | R | A | Q | 278 | Arvicanthis niloticus |
| XP_008835804.1 (+) | 1 | P | G | L | A | E | L | R | R | R | A | Q | 278 | Nannospalax galili |
| XP_027259276.1 (+) | 1 | P | C | L | Q | E | L | R | H | R | V | E | 278 | Cricetulus griseus |
| XP_051054380.1 (+) | 1 | P | S | L | R | E | L | R | S | R | A | V | 278 | Phodopus roborovskii |
| XP_037062288.1 (+) | 1 | P | G | L | A | E | L | R | R | R | A | E | 287 | Peromyscus leucopus |
| XP_059110032.1 (+) | 1 | P | G | L | T | E | L | R | R | R | A | E | 278 | Peromyscus eremicus |
| XP_036034348.1 (+) | 1 | P | G | L | A | E | L | R | R | R | A | E | 278 | Onychomys torridus |
| XP_038204332.1 (+) | 1 | P | G | L | R | E | L | R | R | R | A | E | 278 | Arvicola amphibius |
| XP_050001142.1 (+) | 1 | P | G | L | R | E | L | R | R | R | A | E | 278 | Alexandromys fortis |
| XP_041523764.1 (+) | 1 | P | G | L | R | E | L | R | R | R | A | E | 278 | Microtus oregoni |
| XP_005361967.1 (+) | 1 | P | G | L | R | E | L | R | R | R | A | E | 278 | Microtus ochrogaster |
| XP_057646643.1 (+) | 1 | P | G | L | R | E | L | R | R | R | A | E | 278 | Chionomys nivalis |
| XP_048276061.1 (+) | 1 | P | G | L | R | E | L | R | R | R | A | E | 278 | Myodes glareolus |
| XP_003480127.1 (+) | 1 | P | G | L | A | E | L | R | R | R | A | R | 278 | Cavia porcellus |
| XP_004642417.1 (+) | 1 | P | G | L | A | E | L | R | R | R | A | L | 278 | Octodon degus |
| XP_010639616.1 (+) | 1 | P | G | L | A | E | L | R | R | L | A | R | 283 | Fukomys damarensis |
| XP_021580607.1 (+) | 1 | K | C | G | I | D | L | I | H | Y | L | F | 279 | Ictidomys tridecemlineatus |
| XP_026246591.1 (+) | 1 | P | S | L | A | E | L | R | R | R | A | R | 278 | Urocitellus parryii |
| XP_027788722.1 (+) | 1 | P | S | L | A | E | L | R | R | R | A | R | 278 | Marmota flaviventris |
| XP_046316516.1 (+) | 1 | P | S | L | A | E | L | R | R | R | A | R | 278 | Marmota monax |
| XP_047378799.1 (+) | 1 | P | S | L | A | E | L | R | R | R | A | R | 278 | Sciurus carolinensis |
| XP_045834346.1 (+) | 1 | P | S | L | A | E | L | R | R | R | A | R | 278 | Meles meles |
| XP_032738523.1 (+) | 1 | P | S | L | A | E | L | R | R | R | A | R | 278 | Lontra canadensis |
| XP_047582481.1 (+) | 1 | P | S | L | A | E | L | R | R | R | A | R | 278 | Lutra lutra |
| XP_044101471.1 (+) | 1 | P | S | L | A | E | L | R | R | R | A | R | 278 | Neogale vison |
| XP_032172503.1 (+) | 1 | P | S | L | A | E | L | R | R | R | A | R | 278 | Mustela erminea |
| XP_059250626.1 (+) | 1 | P | S | L | A | E | L | R | R | R | A | R | 278 | Mustela nigripes |
| XP_059022341.1 (+) | 1 | P | S | L | A | E | L | R | R | R | A | R | 278 | Mustela lutreola |
| XP_030159875.1 (+) | 1 | P | A | L | E | E | L | R | R | Q | A | R | 136 | Lynx canadensis |
| XP_046929936.1 (+) | 1 | P | A | L | E | E | L | R | R | Q | A | R | 278 | Lynx rufus |
| XP_043457080.1 (+) | 1 | P | A | L | A | E | L | R | R | Q | A | R | 278 | Prionailurus bengalensis |
| XP_047697695.1 (+) | 1 | P | A | L | A | E | L | R | R | Q | A | R | 278 | Prionailurus viverrinus |
| XP_045311086.1 (+) | 1 | G | G | A | L | L | M | T | E | R | T | L | 311 | Leopardus geoffroyi |
| XP_023104331.1 (+) | 1 | P | A | L | A | E | L | R | R | Q | A | R | 278 | Felis catus |
| XP_049489307.1 (+) | 1 | P | A | L | A | E | L | R | R | Q | A | R | 278 | Panthera uncia |
| XP_042779429.1 (+) | 1 | P | A | L | A | E | L | R | R | Q | A | R | 278 | Panthera leo |
| XP_025727222.1 (+) | 1 | P | S | L | A | E | L | R | R | R | A | R | 278 | Callorhinus ursinus |
| XP_027967096.1 (+) | 1 | P | S | L | A | E | L | R | R | R | A | R | 278 | Eumetopias jubatus |
| XP_027436090.1 (+) | 1 | P | S | L | A | E | L | R | R | R | A | R | 278 | Zalophus californianus |
| XP_021543855.1 (+) | 1 | P | G | L | A | E | L | R | R | R | A | R | 278 | Neomonachus schauins... |
| XP_035944826.1 (+) | 1 | P | S | L | A | E | L | R | R | R | A | R | 278 | Halichoerus grypus |
| XP_006734307.1 (+) | 1 | P | G | L | A | E | L | R | R | R | A | R | 238 | Leptonychotes weddellii |
| XP_034867127.1 (+) | 1 | P | G | L | A | E | L | R | R | R | A | R | 276 | Mirounga leonina |
| XP_045752450.1 (+) | 1 | P | G | L | A | E | L | R | R | R | A | R | 278 | Mirounga angustirostris |
| XP_032277873.1 (+) | 1 | P | S | L | A | E | L | R | R | R | A | R | 278 | Phoca vitulina |
| XP_025333615.1 (+) | 1 | P | S | L | A | E | L | R | R | R | A | R | 278 | Canis lupus dingo |
| XP_055174117.1 (+) | 1 | P | S | L | A | E | L | R | R | R | A | R | 278 | Nyctereutes procyonoides |
| XP_041624832.1 (+) | 1 | P | S | L | A | E | L | R | R | R | A | R | 278 | Vulpes lagopus |
| XP_040481671.1 (+) | 1 | P | S | L | A | E | L | R | R | R | A | R | 278 | Ursus maritimus |
| XP_045644983.1 (+) | 1 | P | S | L | A | E | L | R | R | R | A | R | 278 | Ursus americanus |
| XP_026372210.1 (+) | 1 | P | S | L | A | E | L | R | R | R | A | R | 278 | Ursus arctos |
| XP_057593096.1 (+) | 1 | P | S | L | A | D | L | R | R | R | A | G | 286 | Hippopotamus amphibi... |
| XP_065758979.1 (+) | 1 | P | S | L | A | E | L | R | R | R | A | R | 282 | Muntiacus reevesi |
| XP_043339620.1 (+) | 1 | P | S | L | A | E | L | R | R | R | A | R | 281 | Cervus canadensis |
| XP_043735945.1 (+) | 1 | P | S | L | A | E | L | R | R | R | A | R | 281 | Cervus elaphus |
| XP_060979359.1 (+) | 1 | P | S | L | A | E | L | R | R | R | A | L | 288 | Dama dama |
| XP_020726945.1 (+) | 1 | P | S | L | T | E | L | R | R | R | A | R | 281 | Odocoileus virginianus t... |
| XP_027416865.1 (+) | 1 | P | S | L | A | E | L | R | R | R | A | G | 282 | Bos indicus x Bos taurus |
| XP_061294822.1 (+) | 1 | P | S | L | A | E | L | R | R | R | A | G | 282 | Bos javanicus |
| XP_055402063.1 (+) | 1 | P | S | L | A | E | L | R | R | R | A | G | 282 | Bubalus kerabau |
| XP_025121132.1 (+) | 1 | P | S | L | A | E | L | R | R | R | A | G | 282 | Bubalus bubalis |
| XP_040117221.1 (+) | 1 | P | S | L | A | E | L | R | R | R | T | G | 282 | Oryx dammah |
| XP_052507892.1 (+) | 1 | P | S | L | A | E | L | R | R | R | T | G | 282 | Budorcas taxicolor |
| XP_055287691.1 (+) | 1 | P | S | L | A | E | L | R | R | R | A | R | 281 | Moschus berezovskii |
| XP_047639814.1 (+) | 1 | P | S | L | A | E | L | R | R | R | T | R | 275 | Phacochoerus africanus |
| XP_020944969.1 (+) | 1 | P | S | L | A | E | L | R | R | R | A | R | 275 | Sus scrofa |
| XP_032326070.1 (+) | 1 | P | S | L | A | E | L | R | R | R | V | R | 282 | Camelus ferus |
| XP_031297428.2 (+) | 1 | P | S | L | A | E | L | R | R | R | V | R | 282 | Camelus dromedarius |
| XP_024598337.1 (+) | 1 | A | V | L | I | A | T | G | L | D | Q | R | 326 | Neophocaena asiaeorie... |
| XP_059983478.1 (+) | 1 | V | V | L | I | E | L | L | I | A | S | E | 328 | Lagenorhynchus albiros... |
| XP_026983814.1 (+) | 1 | V | V | L | I | E | L | L | I | A | S | E | 326 | Lagenorhynchus obliqui... |
| XP_059850685.1 (+) | 1 | P | S | L | A | E | L | R | R | R | A | G | 283 | Delphinus delphis |

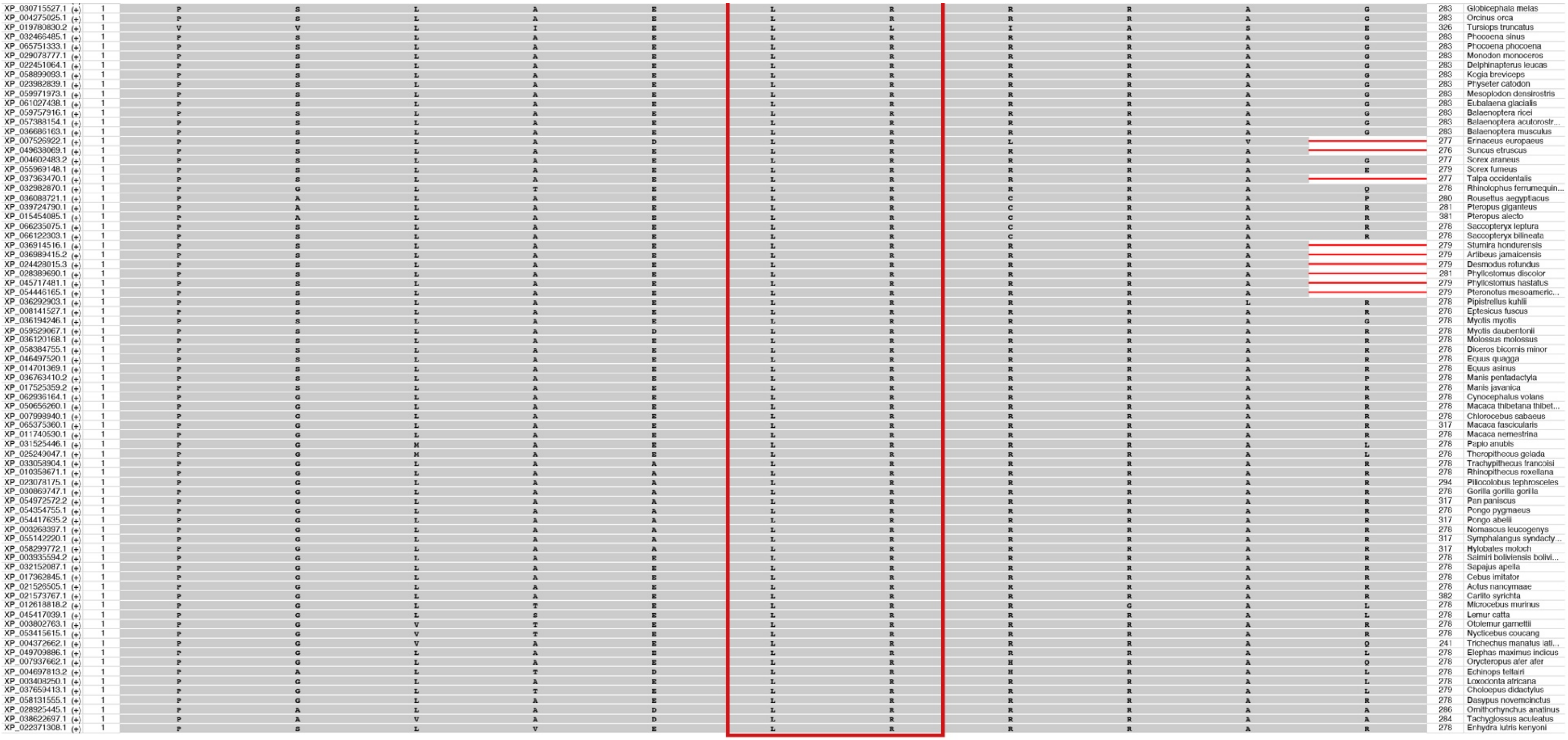

**S1 Figure. Amino acid alignment of p.26 to p.36 of TTPA**

The highly conserved p.L31 and p.R32 amino acids are boxed in red. These amino acids are conserved in 190 mammalian species.
