# Supplementary material for: A TTPA deletion is associated with retinopathy with vitamin E deficiency in the English Cocker Spaniel dog
Source: G3 (Bethesda). 2025 Jan 28;15(4):jkaf016. doi: 10.1093/g3journal/jkaf016 (PMC12005162; doi:10.1093/g3journal/jkaf016)
Supplement: jkaf016_Supplementary_Data [file jkaf016_supplementary_data.zip › S1_Table_G3-2024-405446.pdf]

**S1 Table – List of RVED-affected or suspected cases**

| | Age diagnosis (yrs) | VIT E results (umol/l) | Neurological signs |
|---|---|---|---|
| RVED-affected | 2.41 | 3.3 | |
| RVED-suspected | 2.75 | | |
| RVED-affected | 3 | 1 | |
| RVED-affected | 3 | 1.9 | Seizures |
| RVED-affected | 3 | 2.8 | |
| RVED-suspected | 3 | | |
| RVED-affected | 3.17 | 0 | Hindlimb proprioceptive deficit |
| RVED-affected | 4 | 0.8 | |
| RVED-affected | 4 | 1.6 | |
| RVED-affected | 4 | 10.7 | |
| RVED-affected | 4.42 | 13.28 | |
| RVED-suspected | 4.75 | | |
| RVED-affected | 5 | 6.08 | |
| RVED-affected* | 5 | 6.5 | |
| RVED-suspected | 5 | | |
| RVED-suspected | 5 | | |
| RVED-suspected | 5 | | |
| RVED-affected | 5.58 | 4.8 | |
| RVED-affected | 6 | 2.8 | |
| RVED-affected | 6 | 13.7 | |
| RVED-affected | 6 | 17.7 | |
| RVED-suspected | 6.25 | | |
| RVED-affected | 6.5 | 11.3 | |
| RVED-affected | 6.67 | 0.9 | Hindlimb ataxia / marked hindlilmb proprioceptive defecit |
| RVED-affected | 7 | 3.8 | |
| RVED-affected | 7 | 13.5 | Facial nerve paresis |
| RVED-suspected | 8 | | |
| RVED-affected | 8.3 | 2.175 | |
| RVED-suspected | 8.5 | | |
| RVED-affected | 10 | 0.1 | |
| **MEAN** | **5.28** | **5.65** | |
| **MEDIAN** | **5** | **3.3** | |

Age of diagnosis and plasma $\alpha$-Toc concentrations are illustrated.
RVED-affected* is an English Cocker Spaniel/Cavalier King Charles Spaniel cross.
