# Supplementary material for: A TTPA deletion is associated with retinopathy with vitamin E deficiency in the English Cocker Spaniel dog
Source: G3 (Bethesda). 2025 Jan 28;15(4):jkaf016. doi: 10.1093/g3journal/jkaf016 (PMC12005162; doi:10.1093/g3journal/jkaf016)
Supplement: jkaf016_Supplementary_Data [file jkaf016_supplementary_data.zip › S2_Table_G3-2024-405446.pdf]

S2 Table – Primers used for Sanger sequencing and AFLP of *TTPA* deletion

**Primer Name**	**Primer Sequence**	**Product Size**
Sanger Sequencing Primers		
Sanger_F	CCAGCTCCAGGTCGAAGTC	WT=352bp<br>MUT=250bp
Sanger_R	TGACCCGGGAAAGGAAAGG	
Amplified fragment length polymorphism primers		
Forward_Tailed	TGACCGGCAGCAAAATTGCCAGCTCCAGGTCGAAGTC	MUT=268bp<br>WT=359bp*
Reverse	TGACCCGGGAAAGGAAAGG	
FAM_Tail	TGACCGGCAGCAAAATTG	

* The wild-type fragment size is varied as there are common insertions in the intergenic region upstream of *TTPA*
